# Implementing evidence-based practices in the care of infants with bronchiolitis in Australasian acute care settings: study protocol for a cluster randomised controlled study

**DOI:** 10.1186/s12887-018-1187-7

**Published:** 2018-07-06

**Authors:** Libby Haskell, Emma J. Tavender, Catherine Wilson, Sharon O’Brien, Franz E. Babl, Meredith L. Borland, Liz Cotterell, Tibor Schuster, Francesca Orsini, Nicolette Sheridan, David Johnson, Ed Oakley, Stuart R. Dalziel, on behalf of PREDICT

**Affiliations:** 1 Children’s Emergency Department, Starship Children’s Hospital, Private Bag 92024, Auckland, 1142 New Zealand; 2 University of Auckland, Auckland, New Zealand; 3 Murdoch Childrens Research Institute, Melbourne, Australia; 4 University of Melbourne, Melbourne, Australia; 5 Princess Margaret Hospital for Children, Perth, Australia; 6 The Royal Children’s Hospital, Melbourne, Australia; 7 Department of Paediatrics, University of Melbourne, Melbourne, Australia; 8 Divisions of Paediatrics and Emergency Medicine, School of Medicine, University of Western Australia, Perth, Australia; 9 Armidale Rural Referral Hospital, Armidale, NSW Australia; 10 University of New England, Armidale, NSW Australia; 11 Melbourne Children’s Trials Centre, Melbourne, Australia; 12 Massey University, Auckland, New Zealand; 13 Cumming School of Medicine, University of Calgary, Calgary, Canada; 14 Paediatric Research in Emergency Departments International Collaborative, Melbourne, Australia

**Keywords:** Bronchiolitis, Cluster trial, Knowledge translation strategies, Acute care, Implementation

## Abstract

**Background:**

Bronchiolitis is the most common reason for admission to hospital for infants less than one year of age. Although management is well defined, there is substantial variation in practice, with infants receiving ineffective therapies or management. This study will test the effectiveness of tailored, theory informed knowledge translation (KT) interventions to decrease the use of five clinical therapies or management processes known to be of no benefit, compared to usual dissemination practices in infants with bronchiolitis. The primary objective is to establish whether the KT interventions are effective in increasing compliance to five evidence based recommendations in the first 24 h following presentation to hospital. The five recommendations are that infants do not receive; salbutamol, antibiotics, glucocorticoids, adrenaline, or a chest x-ray.

**Methods/design:**

This study is designed as a cluster randomised controlled trial. We will recruit 24 hospitals in Australia and New Zealand, stratified by country and provision of tertiary or secondary paediatric care. Hospitals will be randomised to either control or intervention groups. Control hospitals will receive a copy of the recent Australasian Bronchiolitis Guideline. Intervention hospitals will receive KT interventions informed by a qualitative analysis of factors influencing clinician care of infants with bronchiolitis. Key interventions include, local stakeholder meetings, identifying medical and nursing clinical leads in both emergency departments and paediatric inpatient areas who will attend a single education train-the-trainer day to then deliver standardised staff education with the training materials provided and coordinate audit and feedback reports locally over the study period. Data will be extracted retrospectively for three years prior to the study intervention year, and for seven months of the study intervention year bronchiolitis season following intervention delivery to determine compliance with the five evidence-based recommendations. Data will be collected to assess fidelity to the implementation strategies and to facilitate an economic evaluation.

**Discussion:**

This study will contribute to the body of knowledge to determine the effectiveness of tailored, theory informed interventions in acute care paediatric settings, with the aim of reducing the evidence to practice gaps in the care of infants with bronchiolitis.

**Trial registration:**

Australian New Zealand Clinical Trials Registry ACTRN12616001567415 (retrospectively registered on 14 November 2016).

## Background

Knowledge translation (KT) in emergency medicine is a relatively new field, and in paediatric emergency medicine (PEM) is in its infancy. There have been several trials assessing the effectiveness of various strategies for KT in emergency medicine and in PEM, although none of these were conducted in Australia or New Zealand [1, 2]. Approaches to KT are varied and, it is likely that barriers and challenges experienced in one region or country are not necessarily experienced in another, resulting in the need to contextualise KT evidence to local environments. A recent systematic review of KT studies in PEM concluded that more optimal study designs with explicit descriptions of implementation were needed to enhance our understanding of how best to translate evidence in to practice [3].

Bronchiolitis is an appropriate condition for testing the effectiveness of KT implementation strategies for a number of reasons; 1. It is an extremely common disease that is seen in small rural hospitals as well as in tertiary paediatric centres [4–6]; 2. It is the most common reason for admission to hospital for infants aged < 1 year. In New Zealand, there are > 70 admissions/1000 infants. Māori (relative risk (RR) 3.0), Pacific (RR 4.3), and those living in the most deprived quintile (RR 4.7) are at most risk [7, 8]; In Australia; bronchiolitis accounts for 56% of all admissions of infants aged less than one year [9]; 3. Hospitalisation is the primary determinant of health care expenditures for the disease [10]; 4. Management is well defined [11, 12]; and necessitates supportive care of oxygen and supplemental hydration with medical and nursing involvement [13, 14]; 5. Substantial variations in practice have been shown to occur [15]. Therefore, effective KT implementation strategies should lower unnecessary health care interventions, improve patient care and allow reallocation of healthcare funds to other areas.

The Paediatric Research in Emergency Departments International Collaborative (PREDICT) network [16] is well placed to undertake this study having completed a multi-centre randomised controlled trial (RCT) of intravenous versus nasogastric fluid replacement in children admitted with bronchiolitis [13, 14, 17–19]. As part of this trial, data were collected on > 3800 admissions for bronchiolitis, over three years from seven Australasian sites. These data show that five therapies and management processes, for which there is high-level evidence that they are ineffective, were used at least once in 27 to 48% of bronchiolitis admissions [20]. Ineffective interventions included inhaled salbutamol, inhaled adrenaline, oral glucocorticoids, antibiotics and chest x-ray.

Having identified both inappropriate care and variation in care, an Australasian Bronchiolitis Guideline for the management of bronchiolitis was developed, providing evidence and recommendations for emergency department (ED) and paediatric inpatient care, with widespread stakeholder endorsement [21]. Guidelines can be formally defined as “systematically developed statements to assist practitioners and patient decisions about appropriate care for specific clinical circumstances” [22]. It is recognised that to effectively manage conditions there needs to be agreement from all specialty groups within individual hospitals involved in care for the condition of interest. Thus, the Australasian Bronchiolitis Guideline was developed using a consensus process across both countries utilising craft groups and specialists from both the ED and paediatric inpatient units involved in management of infants with bronchiolitis. Recommendations from this guideline will be implemented using tailored, theory informed KT interventions in this study as it is now accepted that simply providing such a guideline to clinicians is insufficient to significantly change practice [23].

Using a theoretical approach to develop KT interventions is increasing considered to be best practice [24]. Tailored interventions (interventions planned following investigation into the factors that influence practice and reasons for resisting practice change) are more likely to improve practice than no intervention [25]. Cochrane Effective Practice and Organisation of Care systematic reviews have shown that: Interventions consistently showing effectiveness include audit and feedback [26], interactive educational meetings, educational outreach visits, reminders (either manual or computerised) and multifaceted interventions (defined as a minimum of two combined interventions); Interventions that have shown some improvement include the use of local champions and local consensus processes and patient-mediated interventions; Interventions that consistently show little or no effect are didactic educational meetings and educational materials such as those distributed for recommendations of clinical care, including practice guidelines, electronic publications and audio-visual materials [27, 28]. However, there is little evidence on the effectiveness of interventions in PEM settings.

We are therefore undertaking a cRCT to determine if tailored, theory informed KT interventions are effective in improving evidence-based practice in Australasian paediatric acute care settings.

## Methods

### Aim

The aim of the study is to test the effectiveness of tailored, theory informed KT interventions at increasing the uptake of five evidence-based recommendations from the Australasian Bronchiolitis Guideline [14]. The five key recommendations from the guideline are that infants under one year of age with bronchiolitis who present to hospital receive none of the following: 1. Salbutamol; 2. Antibiotics; 3. Glucocorticoids; 4. Adrenaline; 5. A chest x-ray (see Table 1).

**Table 1:** Key clinical recommendations from the Australasian Bronchiolitis Guideline

Clinical intervention	NHMRC strength of recommendation	GRADE quality of evidence	Guideline recommendation
Salbutamol	A	Strong	Do not administer salbutamol
Antibiotics	B	Conditional	Do not use antibiotics
Glucocorticoids	B	Strong	Do not administer systemic or local glucocorticoids (nebulised, oral, intramuscular or intravenous)
Adrenaline	B	Strong	Do not administer adrenaline (nebulised, intramuscular or intravenous)
Chest x-ray	D	Conditional	Chest x-ray is not routinely indicated

*NHMRC* National Health and Medical Research Council, *GRADE* Grading of Recommendations, Assessment, Development and Evaluations

### Research objectives

The primary objective is to determine the effectiveness of tailored, theory informed KT interventions versus passive dissemination of a bronchiolitis guideline in decreasing use of therapies and management processes known to be of no benefit in infants with bronchiolitis.

Secondary objectives include evaluating the effectiveness of the interventions at decreasing duration of hospital stay for infants with bronchiolitis and determining their relative effectiveness in tertiary paediatric and secondary hospitals. In addition we will also quantify health care costs (including cost associated with guideline development and implementation) and measure change in median medication doses.

Integral to our primary objective is the need to evaluate the fidelity of the KT interventions and assess receipt, delivery and acceptability of the KT interventions by conducting a process evaluation.

### Design

**Fig. 1:**
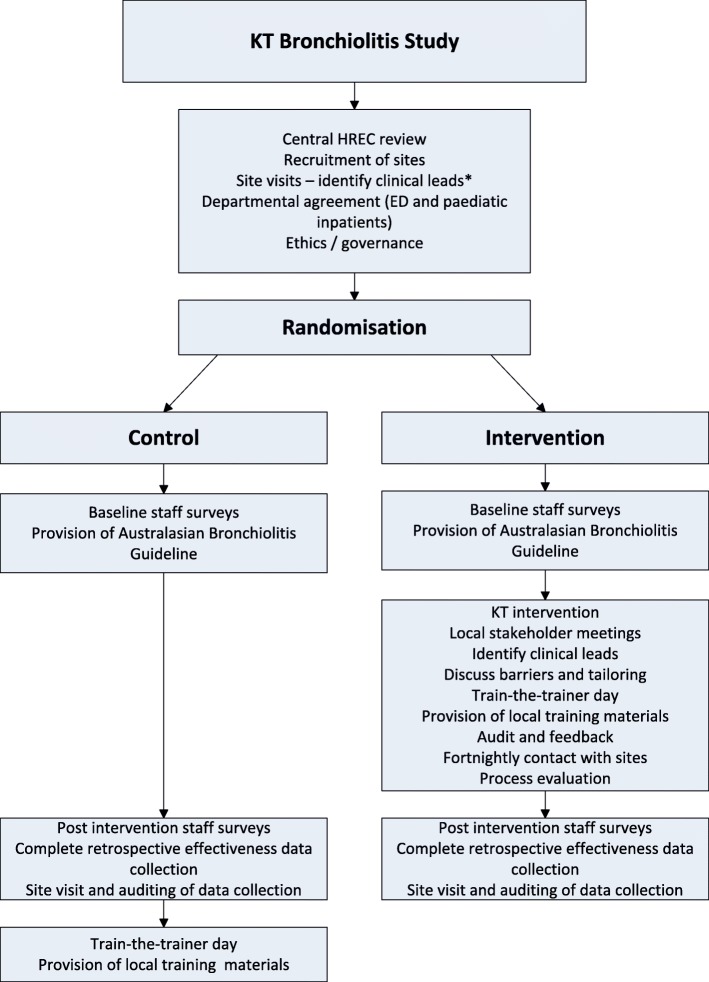
KT study process design*Site visit to include: meeting clinical directors, discussion re study requirements, ethics and the departmental agreement. KT = Knowledge Translation. HREC = Health Research Ethics Committee

This is a multi-centre cRCT where the hospitals, ED and paediatric inpatient staff members, represent the units of randomisation. A randomised design is advantageous in evaluating the effectiveness of an intervention since bias is minimised when estimating intervention effects compared with other study designs [29, 30]. Clusters have been chosen for the following reasons: 1) the intervention is targeted to the staff involved in the care of infants with bronchiolitis, 2) the hospitals represent distinct non-independent patient populations in both geographical areas and levels of paediatric service provision, and 3) a RCT involving randomisation at the level of the patient is impractical [31, 32]. The study design is outlined in Fig. 1.

### Recruitment and eligibility

#### Recruitment of hospitals and inclusion/exclusion criteria

Hospitals will be identified in two ways; through the members of the PREDICT research network and from the Australasian College for Emergency Medicine ED Directory list of 24 h Australasian EDs (regional and metropolitan) until a total of 24 hospitals are recruited (six New Zealand and 18 Australian hospitals), ensuring selection across different states and hospitals that provide different levels of paediatric care. Hospitals who have principal or co-investigators in this study, or who had clinicians with significant involvement in the writing of the Australasian Bronchiolitis Guideline will be excluded to reduce the potential risk of bias in the study results.

Following initial contact, a recruitment pack detailing the proposed study will be sent to both clinical directors and nursing managers of ED and paediatric inpatient areas. A recruitment meeting will follow (via telephone or face-to-face) to discuss details and logistics of the study (with expectations of hospitals as well as the study team detailed). A baseline checklist will be completed to quantify annual bronchiolitis presentation numbers.

**Inclusion criteria for hospitals:**

- Have a confirmed ED census of > 135 bronchiolitis presentations per year.
- Be willing to participate and abide by the randomisation schedule (either control or intervention).
- A signed department agreement by both ED and paediatric inpatient clinical directors.
- Have the ability to collect the required retrospective patient data from clinical notes.

**Exclusion criteria for hospitals:**

- Inability to audit clinical notes.
- Be averse to participating if randomised to the control arm.
- Those hospitals having clinicians who are principal or co-investigators in this study or had significant involvement in the writing of the Australasian Bronchiolitis Guideline.

### Randomisation and allocation concealment

Randomisation of hospitals into either intervention or control groups will be completed using randomisation Stata 14.2 statistical software, by a statistician not affiliated with the study. Stratification will be undertaken by country (Australia and New Zealand) as well as whether the hospital is a tertiary or secondary paediatric hospital. A hospital will be classified as tertiary if they have a dedicated paediatric intensive care unit.

### Blinding

Due to the nature of the intervention, it will not be possible to blind staff members involved in the study to group allocation. This is a potential threat to the validity of the study. Communication between intervention and control groups will be discouraged but still may occur.

Ideally hospitals will use local data collectors not associated with patient care in ED or paediatric inpatient areas and who are not aware of study outcomes. The data collection training will focus on the operational aspects of the study. A percentage of data collection at each site will be independently audited to confirm the authenticity of the data.

### Knowledge translation interventions

#### Intervention groups

Hospital intervention groups will receive tailored, theory informed KT interventions. A stepped approach will be followed to develop the interventions including: identifying five key evidence-based recommendations from the bronchiolitis guideline as well as using findings from a previous qualitative study where clinicians were interviewed to identify factors perceived to influence the management of infants with bronchiolitis. The interview schedule was developed and interviews analysed using the Theoretical Domains Framework (TDF) which describes a comprehensive structure of 14 theoretical domains from 33 behaviour change theories and 128 constructs. The TDF has demonstrated strong explanatory and predictive powers across a number of healthcare settings with helpfulness in advising interventions to improve practice change [33, 34]. This framework has recently been successfully trialled in an Australian ED environment to understand factors influencing the management of mild traumatic brain injury in adults to then guide intervention development [35]. Thematic analysis findings from the qualitative interviews will be mapped to behaviour change techniques with interventions being developed to address influencing factors. Table 2 summarises the intervention components of this protocol.

**Table 2:** Planned delivery of the intervention

All interventions	Intervention site	Control site
Electronic and printed copy of complete Australasian Bronchiolitis Guideline	✓	✓
Electronic and printed copy of summarised bedside clinical Australasian Bronchiolitis Guideline	✓	✓
Multidisciplinary key stake holder meeting to create organisational buy-in	✓	
Identification of up to four clinical leads (medical and nursing) from ED and paediatric inpatient areas	✓	
One day train-the-trainer workshop for clinical leads	✓	
Provision of KT materials for local training • Educational power points • Fact sheets • Posters • Parent / caregiver information sheet	✓	
Monthly audit and feedback site reports	✓	
KT study manual	✓	
Support for clinical leads during intervention period by key research group contact	✓	

*ED* emergency department, *KT* knowledge translation

#### Control groups

Hospitals in the control group will receive an electronic and printed copy of the complete Australasian Bronchiolitis Guideline as well as the summarised bedside clinical version. At the end of the study (after effectiveness data collection has occurred), control hospitals will be offered a one day training day which will cover similar content to that which the intervention groups received.

#### Research outcomes

Primary outcome

Compliance or non-compliance for each patient presentation with the guideline during the first 24 h following presentation to ED (acute care period), with regards to the use of five key therapies and management processes known to have no benefit (salbutamol, antibiotics, glucocorticoids, adrenaline and chest x-ray).

Secondary outcomes

1. Compliance or non-compliance for each patient presentation with the guideline with regards to the use of key therapies and management processes known to have no benefit (salbutamol, antibiotics, glucocorticoids, adrenaline and chest x-ray):
  - While in ED.
  - While an inpatient.
  - During total hospitalisation.
2. Compliance or non-compliance for each patient presentation with guideline recommendations during the first 24 h following presentation to the ED (acute care period) with regards to the use of:
  - Salbutamol.
  - Antibiotics.
  - Glucocorticoids.
  - Adrenaline.
  - Chest x-ray.
3. Compliance or non-compliance for each patient presentation with guideline recommendations during their total hospitalisation with regards to use of:
  - Salbutamol.
  - Antibiotics.
  - Glucocorticoids.
  - Adrenaline.
  - Chest x-ray.
4. Process evaluation including measure of receipt, delivery and acceptability.
5. Length of stay.
6. Death and or intensive care admission.
7. Health care costs (including cost associated with guideline development and implementation).
8. Median number of medication doses:
  - In acute care period.
  - During total hospitalisation.

### Identification of study patients

In order to determine the effect of the intervention on clinical practice outcomes, data extraction from a random selection of patient records will be conducted by chart auditors appointed at each site. The numbers of patient records required are:

- 1/05/14–30/04/2016 - Retrospective chart audit (100 patients/year) pre KT interventions (2 years)
- 1/5/16–30/4/17 – Retrospective chart audit (100 patients) - “washout period” during which the Australasian Bronchiolitis Guideline was released (1 year)
- 1/05/17–30/11/17 – Retrospective chart audit (155 patients) post KT intervention (7 months)

### Inclusion/exclusion criteria

Patients will be eligible for data extraction if they are aged less than 12 months (at time of presentation), AND have a recorded diagnosis of bronchiolitis on discharge from ED to home, OR a diagnosis of bronchiolitis on discharge from the paediatric inpatient area AND a recorded diagnosis of bronchiolitis in ED. It is important that admitted infants are only included in the data analysis where both the ED and paediatric inpatient clinicians believed they had bronchiolitis and therefore should have been managed as such. If the patient was admitted directly to the paediatric inpatient area without being seen in ED (as occurs in some hospitals), and was discharged with a recorded diagnosis of bronchiolitis, they will be included. There will be no exclusion on the basis of co-morbidities, transfer from other health care facilities or representation with bronchiolitis.

### Staff survey

Medical and nursing staff from ED and paediatric inpatient areas in the intervention and control groups will complete two surveys (at baseline and post intervention), to explore factors that may influence how they manage infants with bronchiolitis. Inclusion criteria for staff members completing surveys will be: 1) current ED or general paediatric inpatient area employee; 2) on active practice roster; 3) registered nurse, enrolled nurses, or 4) registrars, house officers (or equivalent) or consultants. Exclusion criteria for staff members completing surveys will be: 1) students or interns; 2) clinicians not currently engaged in clinical practice; 3) agency or bank staff (nurses), or locums (medical). Eligible staff members identified by a research identification number only, will be randomly selected to receive a staff survey by the central study team. The site clinical lead will organise delivery of an invitation letter and information form with the survey to the selected staff members. Consent will be implied if a completed survey is returned to the central research team. Staff who decline or are unavailable to participate will be replaced by another randomly selected person at a similar level of practice.

10 staff members from each departmental group will be asked to complete a survey e.g. 10 ED nurses, 10 ED medical staff, 10 paediatric inpatient nurses and 10 paediatric inpatient medical staff. Those who completed baseline surveys will be sent post intervention surveys wherever possible. Additional staff will be randomly selected to complete a survey if baseline staff are lost to follow-up at the post intervention stage.

## Data quality assurance

### Patient data

Data collection and accurate documentation will be the responsibility of the site study staff, under the supervision of the site principal investigator. A site manual will be provided to each clinical lead which details data collection processes for the study.

Infants with International Classification of Diseases-9 or 10 (ICD-9 or ICD-10) codes related to bronchiolitis for the respective time periods of data collection will be identified. From this list, the research group will randomly select the required number of infants for each time period for whom data will be extracted for. Trained chart auditors will review all records to ensure eligibility criteria are met. Training will maximise consistency both in identification of patients and minimising selective auditing in addition to attaining consistency in data collection.

**Patient data to be collected:**

- Date of birth
- Sex
- Ethnicity
- ED length of stay
- Disposition from ED
- Length of stay for inpatients
- Length of stay for those admitted to intensive care unit
- Past history
- Salbutamol administration during hospitalisation: number and timing of doses
- Antibiotics administration during hospitalisation: number and timing of doses
- Glucocorticoid administration during hospitalisation: number and timing of doses
- Adrenaline administration during hospitalisation: number and timing of doses
- Chest x-ray during hospitalisation, time taken and chest x-ray report
- Use of supplementary oxygen during hospitalisation
- Use of high flow during hospitalisation

Data will be entered into the Research Electronic Data Capture (REDCap) study database, housed at Murdoch Childrens Research Institute (MCRI), Melbourne, Australia. Each site will maintain an electronic log book containing patient research identification codes to enable data to be re-identified at the site if required.

During site visits, a small sample (10) of clinical notes will be reviewed by the study team in order to formally test reliability and accuracy of data extraction.

### Staff survey data

The survey will explore factors that may influence how clinicians manage infants with bronchiolitis, and site departmental change management, with change measured from baseline (start of study) to post intervention (end of study). De-identified, completed surveys with a site code and study participant number will be returned to the researchers by mail. Clinical leads and data entry staff at each site will not have access to the data. Survey data will be entered into REDCap. Researchers will have access to the data and study participant number, but no identifiable information other than the site code and study participant number.

### Process evaluation data

Process evaluation data will be collected at each intervention site [36] and provide data on the feasibility of the materials, whether they reached the target group, perceptions on the delivery and receipt of materials [37], as well as the fidelity to the intended use of the intervention components. Part of the role of the clinical leads in the intervention arm, will be to ensure information is collected for process evaluation. These data will be entered directly into REDCap or on to an excel spread sheet (which will then be entered in to REDCap), with detail on time involved, numbers of personnel educated, materials used and any information to provide more detail on challenges, successes, and any problems or positive experiences that occurred. Clinical leads will not be able to see any other sites’ data. This information will be complemented by interviews with clinical leads at the end of the study in order to explore and explain any emerging issues. A comprehensive commentary on each intervention used will be created.

#### Sample size

The primary outcome of this study is compliance to the Australasian Bronchiolitis Guideline in regards to the five therapies and management processes known to have no benefit.

The sample size calculation is bound by the assumption of an absolute increase in compliance to the guideline of at least 15% in the intervention arm compared to the control arm in which, according to preliminary data, compliance to the guideline is assumed to be close to 50%. The rationale for selecting an absolute increase in compliance of 15% is that it is both clinically relevant and also justifies the resource associated with delivery of the intervention. A sample size of 1620 infants per arm (3240 in total) is required to provide 82% power (alpha = 0.05) to detect a minimum difference of 15% in compliance with the guideline, allowing for an average intra-class correlation coefficient of 0.055 (based on previous data from seven Australian Hospitals [20]) and an average cluster size of 135 (24 clusters in total).

The sample size calculations took the nature of the outcome (binary variable outcome variable: guideline compliance yes/no) as well as the issue of clustering (heterogeneity in estimated proportions between sites that exceeds variability being explained by random sampling) into account.

#### Effectiveness analysis

The primary analysis will be on an Intention-To-Treat (ITT) basis.

The principal analysis will examine compliance or non-compliance with the guideline, for each individual patient in the acute care period, ED and as an inpatient, with regards to key therapies and management processes known to have no benefit.

A Generalized Linear Mixed Models (GLMM) will be used to estimate the marginal difference in the proportion of bronchiolitis patients treated in accordance with the existing guideline between the study arms. The GLMM approach will employ a logit link function and will include random effect terms for study site. Based on the GLMM, risk differences between the proportions of compliance to the guideline in the two arms with its 95% confidence intervals will be computed. Missing outcome data will be imputed using multiple imputation model.

The details of the primary and secondary statistical analyses will be specified in a separate statistical analysis plan (SAP) which will be finalised before study database lock. The SAP will detail covariates to be considered in the primary analysis model as well as subgroup and sensitivity analyses to be performed. The SAP will also outline the multiple imputation strategy to handle missing data.

#### Economic evaluation

An economic analysis will be carried out with costs associated with the hospital stay. This will include: cost of ED presentation, cost of admission and cost of therapies. Additionally, costs associated with the development of the Australasian Bronchiolitis Guideline and the KT intervention development and implementation will be analysed.

#### Process evaluation

Both quantitative and qualitative data will be gathered to evaluate the process of implementation of the intervention. This data will be analysed to assess fidelity in the delivery of the KT interventions (what was delivered, to whom and how) as well as undertaking a thematic analysis of staff response to open ended questions about the acceptability of the interventions and their perceptions of facilitators and barriers encountered.

### Ethics approval and consent to participate

This study has undergone ethics review and been approved by the Royal Children’s Hospital Human Research Ethics Committee (EC00238), Australia (reference HREC/16/RCHM/84) and the Northern A Health and Disability Ethics Committee, New Zealand (reference 16/NTA/146). Hospitals agreeing to take part will obtain local research governance review. This includes a departmental agreement signed by both the clinical director of ED and inpatients and a data transfer agreement.

### Confidentiality of data

Confidentiality of data will be ensured via the following means: 1) all data collected will be entered in to REDCap – a secure, web-based sever, 2) unique password protected usernames will be provided to REDCap users with different levels of access dependent on the person’s role in the study, 3) patient data entered into REDCap will be de-identified, and 4) staff data returned centrally will also be de-identified.

## Discussion

This study aims to evaluate the implementation of tailored, theory informed KT interventions to improve key evidence-based recommended practices for the management of infants with bronchiolitis in Australasian EDs and paediatric inpatient settings. This study builds upon previous work to assess the effectiveness of tailored interventions in acute settings and use of the TDF [35, 38]. This study will specifically look at whether these interventions can reduce the use of ineffective therapies and management processes for bronchiolitis, a paediatric condition frequently seen in the acute care setting. This study will have implications beyond bronchiolitis management, with improving knowledge of KT in the paediatric acute care setting. We believe that this is the first study to evaluate these five key recommendations.

### Trial status

At the time of submitting this paper, recruitment of 26 sites has occurred with each attaining local ethics and governance requirements. Two more sites were recruited than originally planned, to allow for the possibility of a site withdrawing at a late stage. Randomisation has occurred and KT interventions have been undertaken in intervention sites. Effectiveness data collection has commenced. The trial is registered in the Australian and New Zealand Clinical Trials Registry (ACTRN12616001567415).

## Abbreviations

ACEM: Australasian College of Emergency Medicine
cRCT: Cluster randomised controlled trial
ED: Emergency department
GLMM: Generalized Linear Mixed Models
HDEC: Health and Disability Ethics Committee
ICD: International Classification of Diseases
KT: Knowledge translation
NEAF: National Ethics Application Form
PEM: Paediatric emergency medicine
PREDICT: Paediatric Research in Emergency Departments International Collaborative
REDCap: Research Electronic Data Capture
SAP: Statistical analysis plan
TDF: Theoretical Domains Framework
